# A Fluidically Tunable Metasurface Absorber for Flexible Large-Scale Wireless Ethanol Sensor Applications

**DOI:** 10.3390/s16081246

**Published:** 2016-08-06

**Authors:** Hyung Ki Kim, Dongju Lee, Sungjoon Lim

**Affiliations:** School of Electrical and Electronics Engineering, College of Engineering, Chung-Ang University, 221 Heukseok-dong, Dongjak-gu, Seoul 156-756, Korea; muechu@naver.com (H.K.K.); dongju0721@hanmail.net (D.L.)

**Keywords:** metamaterial, metasurface, absorber, microfluidics, wireless sensor

## Abstract

In this paper, a novel flexible tunable metasurface absorber is proposed for large-scale remote ethanol sensor applications. The proposed metasurface absorber consists of periodic split-ring-cross resonators (SRCRs) and microfluidic channels. The SRCR patterns are inkjet-printed on paper using silver nanoparticle inks. The microfluidic channels are laser-etched on polydimethylsiloxane (PDMS) material. The proposed absorber can detect changes in the effective permittivity for different liquids. Therefore, the absorber can be used for a remote chemical sensor by detecting changes in the resonant frequencies. The performance of the proposed absorber is demonstrated with full-wave simulation and measurement results. The experimental results show the resonant frequency increases from 8.9 GHz to 10.04 GHz when the concentration of ethanol is changed from 0% to 100%. In addition, the proposed absorber shows linear frequency shift from 20% to 80% of the different concentrations of ethanol.

## 1. Introduction

Various chemicals used in modern industry and research are being managed by material safety data sheets (MSDSs) and the Globally Harmonized System (GHS) of classification and labelling of chemicals. In spite of these standards, sometimes unidentified chemicals are generated by complex experimental processes. Some of these chemicals are harmful to the human body. For example, methanol affects the central nervous system and can cause great damage leading to blindness, coma, or death if ingested [1]. Therefore, the detection and quantification process of the chemicals used in a variety of environments is essential.

Permittivity and permeability of a metamaterial can be artificially manipulated by employing conductive patterns. Furthermore, a metasurface can be realized by using 2-dimensional periodic conductive patterns on a planar dielectric substrate. The metasurface can be used as an electromagnetic (EM) wave absorber by using EM resonators such as split ring resonators (SRRs) [2]. In addition, the absorbing scheme relies strongly on having the dielectric slab with metallic wires of a ground plane [3]. The metasurface including EM wave absorber can be realized on a thin film, therefore, it has high flexibility [4,5,6,7]. The resonance frequency of the metasurface is highly sensitive to the inductive and capacitive components of the resonator [8,9]. This feature suggests the possibility of using a metasurface in sensor applications. For example, a metasurface-implemented strain sensor has been introduced [10]. The sensor can sense the strain by measuring resonance frequency shifts. The concept of using metamaterial resonators as thin film sensing devices has been proposed [11,12,13,14]. For instance, the resonant frequency of a split-ring resonator array can be changed by adding solid materials such as silicon nanospheres on the surface [11]. The thin film sensor concept is proposed using the microwave metamaterial resonator in the guided wave although its sensitivity is not demonstrated with materials [12]. Its sensitivity is increased by adding field confining tips to the end pieces of the resonator arms. In additions, sensor applications using metamaterial absorber has been researched in the THz regime [13,14,15,16,17]. For example, the terahertz plasmonic structure is used to enhanced sensing capabilities [15].

Microfluidics is a method for analyzing a fluid by using only a microliter scale of fluid. Therefore, it can reduce the minimum required amount of fluid for analysis as compared with conventional analysis methods [18,19]. In addition, it has the advantage of miniaturization. In recent years, microfluidics has been used in various applications such as bioassays, blood analysis, and controlling manufacturing quality [20,21,22,23,24]. Recent studies on microfluidics have been expanded to fluid-tunable radio frequency (RF) systems and fluid detection microwave systems [24,25]. Most of the studies on RF microfluidics have used fluid as a substitutive dielectric material for microwave resonators, antennas, or transmission lines [26,27,28].

In the present study, we introduce a microfluidic metasurface absorber for flexible large-scale ethanol sensor applications. The proposed metasurface absorber can detect the ethanol concentration from the shifting resonance frequency of the absorber. At a resonance frequency, the metasurface absorbs the incident EM wave energy, so we can determine the ethanol concentration by measuring the absorption frequency. The metasurface is composed of split-ring-cross resonators (SRCRs). We used inkjet printing technology to realize the conductive SRCR patterns on photo paper. Therefore, the proposed metasurface has the advantage of flexibility as compared with conventional printed circuit board (PCB)-based metasurface. In addition, we realized microfluidic channels on a polydimethylsiloxane (PDMS) substrate by using a laser etching technique, so the microfluidic channel also has flexibility. The performance of the proposed flexible absorber has been proved through simulated and measured results.

## 2. Design and Structure

**Figure 1:**
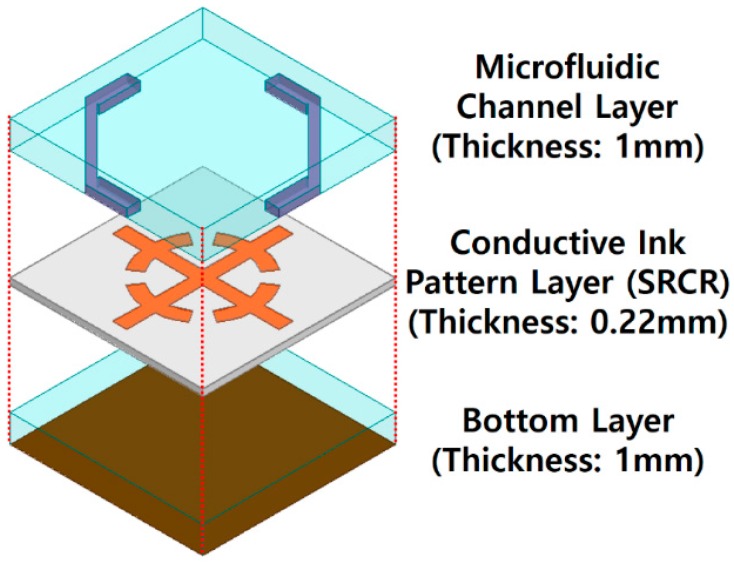
Three-dimensional view of unit cell design.

Figure 1 shows the three-dimensional (3D) view of the final metasurface unit cell design. The proposed metasurface is composed of three layers. The top layer has microfluidic channels on the PDMS substrate. The middle layer has a conductive SRCR pattern inkjet-printed on Kodak premium photo paper. The bottom layer has an additional PDMS substrate in order to minimize transmission coefficient by increasing thickness of substrates. A high absorption rate can be achieved by minimizing both transmission and reflection coefficients. In addition, the bottom side of the metasurface is fully covered with a copper sheet.

**Figure 2:**
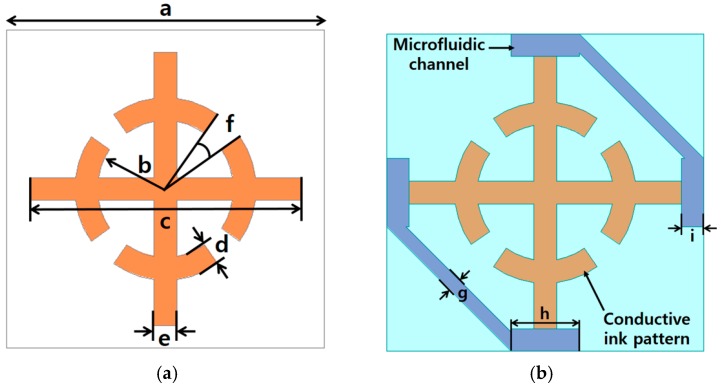
Layout of the proposed metasurface: *a* = 7 mm, *b* = 1.5 mm, *c* = 6 mm, *d* = 0.5 mm, *e* = 0.5 mm, *f* = 20°, *g* = 0.3 mm, *h* = 1.5 mm, *i* = 0.5 mm. (**a**) Without microfluidic channels; (**b**) With microfluidic channels.

The geometry of the proposed metasurface unit cell is shown in Figure 2a. The unit cell is composed of the SRCR because of its simple and symmetric design. Figure 2b shows the layout of the proposed metasurface with microfluidic channels. In order to maximize the frequency shift, the microfluidic channels must be loaded where the electric field is strongly coupled, as illustrated in Figure 2b.

A metasurface shows a frequency-dependent complex number of permittivity and permeability under effective medium approximation [29]. When an EM wave is normally incident on the metasurface, the intrinsic impedance of the metasurface *Z*(*ω*) is determined by the following relation between relative permeability *μ*_r_ and permittivity *ε*_r_ of the metasurface:

(1) $$Z(\omega )=\sqrt{\frac{\mu_{0}\mu_{r}(\omega )}{\varepsilon_{0}\varepsilon_{r}(\omega )}}$$

where the *μ*_0_ and *ε*_0_ are the permeability and permittivity of free space, respectively.

From Equation (1), if we tail *μ*_r_ and *ε*_r_ to the same value, the intrinsic impedance of the metasurface can be matched to that of free space; therefore, reflection waves can be minimized. In addition, the refractive index n of the metasurface has a high value for its imaginary component, and will dissipate the incident EM wave energy. Therefore, a metasurface can achieve a high absorption rate despite its low profile.

The resonance of the SRCR is generated by the inductive and capacitive components of the conductive pattern. A cross wire of the SRCR generates inductance, and a gap at the circular ring and between adjacent unit cells generates capacitance. Capacitance of the SRCR is determined by [30]:

(2) $$C≅\frac{\varepsilon_{eff}10^{-3}}{18\pi }\frac{K(k)}{K\prime (k)}l$$

where the *ε_eff_*, *K(k)*/*K’(k)*, and *l* are the effective dielectric constant, approximate ratio between the elliptic integrals, and width of the capacitive gap, respectively. From Equation (2), the resonant frequency of the SRCR depends on the geometrical dimension as well as the effective dielectric constant. Therefore, in this study, we adapted the microfluidic channel to change the dielectric constant, and thereby sense the resonance frequency shift.

**Figure 3:**
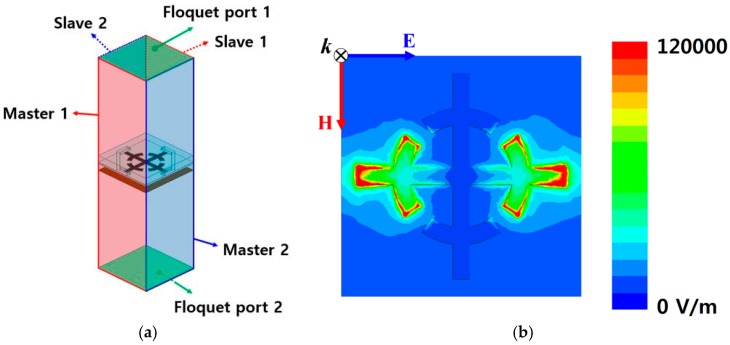
(**a**) Simulation setup for boundary conditions and excitations; (**b**) Magnitude of the electric field distribution of the proposed metasurface without a microfluidic channel.

As the first step in designing the microfluidic channel, we verified the electric field distribution of the SRCR by using a finite-element-method (FEM)-based ANSYS high-frequency structure simulator (HFSS). The proposed unit cell was simulated as an infinite periodic structure. We set up the master/slave boundary conditions and Floquet port excitations for the simulation in Figure 3a. As shown in Figure 3b, a strong electric field is generated around two capacitive gaps between the adjacent unit cells and four gaps of the SRCR. However, it is difficult to load the microfluidic channel on the gaps of the SRR. Therefore, the microfluidic channel is placed on the gap between the adjacent unit cells.

**Figure 4:**
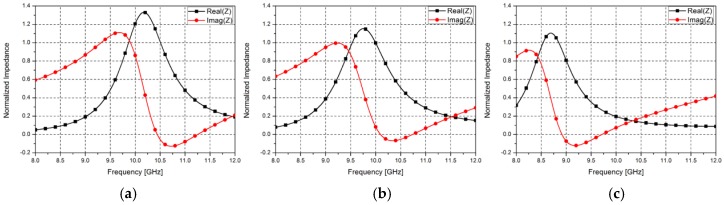
Simulated normalized impedance for (**a**) empty channel state; (**b**) channel filled with ethanol; and (**c**) channel filled with DI water.

Figure 4 shows the simulated impedance of the proposed metasurface after normalizing to the impedance of free space. The proposed metasurface resonates at 10.49 GHz, 10.04 GHz, and 8.9 GHz when the channel is empty, filled with 100% ethanol, and filled with deionized (DI) water, respectively. The complex permittivity of DI water and ethanol are 76 + j21 and 24 + j8.16, respectively. Although their permittivity is dependent on a frequency, we used constant permittivity because an operating frequency band is narrow. At each resonant frequency, the real parts are near unity; therefore, the impedance of the proposed metasurface is matched to that of free space.

**Figure 5:**
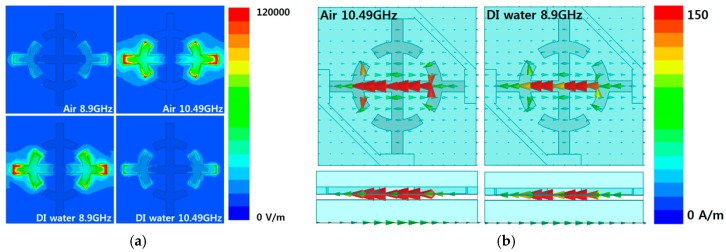
Simulated (**a**) electric field distributions and (**b**) vector current distributions.

To verify the electric and magnetic resonance at the empty-channel state and the DI water-filled state, the electric field distribution and vector current distribution are plotted in Figure 5. The SRCR pattern provides an electric response, as shown in Figure 5a. For the empty-channel state and DI water-filled state, a strong electric field is generated at 10.49 GHz and 8.9 GHz, respectively. Magnetic resonance comes from the anti-parallel current flow, as shown in Figure 5b.

## 3. Results and Discussion

In order to verify the performance of the proposed metasurface absorber, 15 × 15 unit cells were printed three times on Kodak premium photo paper. The relative permittivity, dielectric loss, and thickness of the paper were 2.35, 0.11, and 0.22 mm, respectively. We used a DMP 2800 material printer (Dimatix, Santa Clara, CA, USA) equipped with a Dimatix 10 pL cartridge (DMC-11610). In general, silver and copper nanoparticle inks are available for inkjet-printing technology. Silver nanoparticle ink has higher conductivity than copper nanoparticle ink although silver is more expensive than copper. In this work, the ANP DGP 40LT-15C silver nanoparticle ink is used because of its high conductivity. After printing, the sample was sintered in an oven for 5 min at 180 °C. Through the sintering process, the conductivity of the silver nanoparticle ink increased from 9 × 10^6^ S/m to 1.1 × 10^7^ S/m [31].

**Figure 6:**
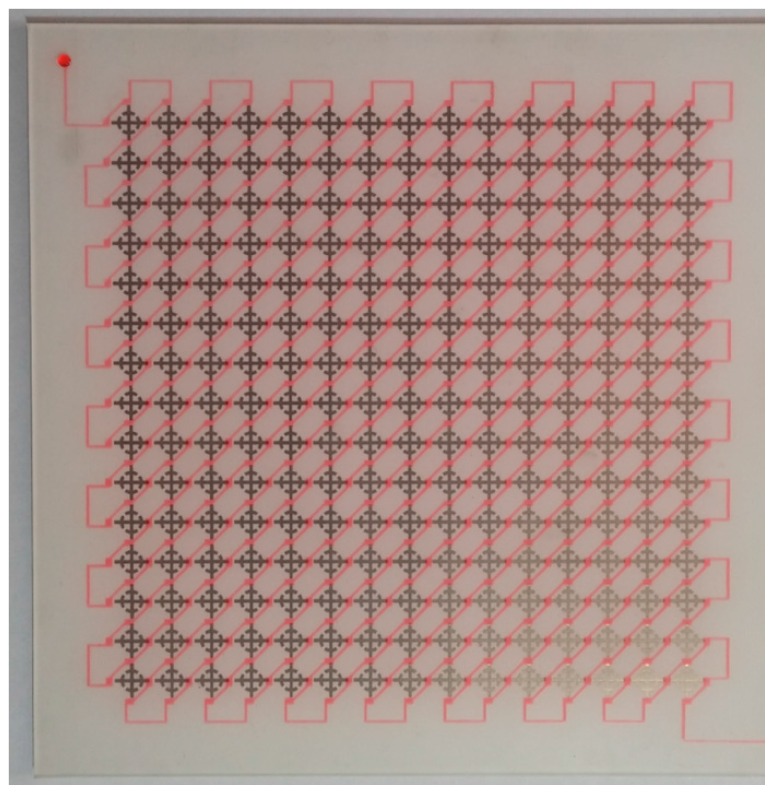
Fabricated prototype sample after injecting DI water. DI water was mixed with red ink in order to clearly see the liquid path.

The top and bottom layer consisted of PDMS. The relative permittivity, dielectric loss, and thickness of PDMS were 4.44, 0.075, and 1 mm, respectively. The microfluidic channel was realized on the PDMS substrate by using a laser etching technique, and the channel depth is 0.3 mm. Each layer was bonded by adhesive film (ARcare^®^ 92561, Adhesives Research, Glen Rock, PA, USA) after a plasma surface treatment. The fabricated prototype sample is shown in Figure 6. To make it easy to check the microfluidic channel, we mixed DI water with red ink and injected it into the channel.

**Figure 7:**
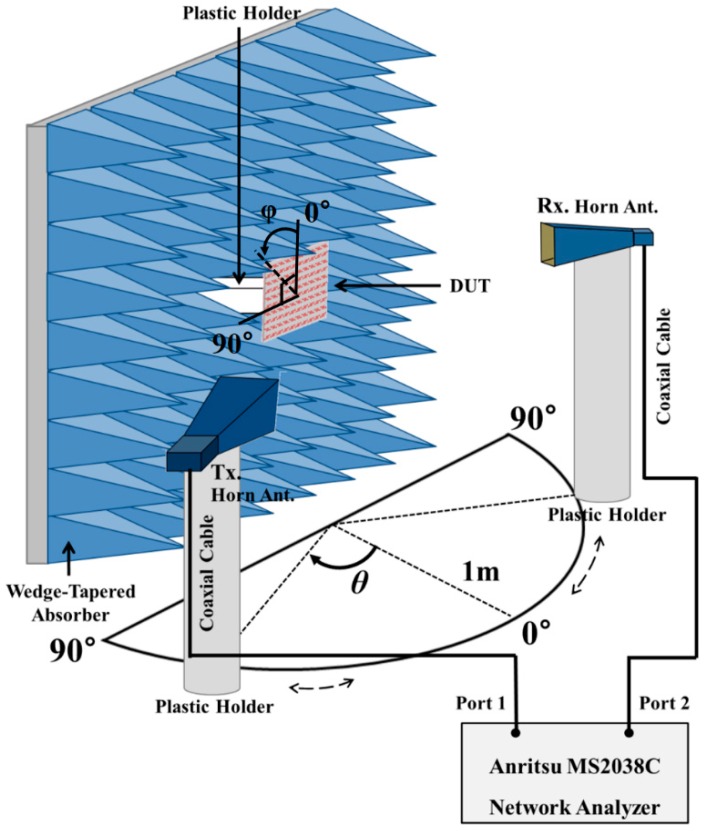
Illustration of the measurement environment.

To investigate the performance of the proposed metasurface absorber, the reflection coefficients were measured using a bistatic radar cross section (RCS) measurement setup [32]. The measurement setup is shown in Figure 7. We measured the S-parameters using an MS2038C vector network analyzer (Anritsu, Morgan Hill, CA, USA). The fabricated sample is surrounded by a wedge-tapered absorber to prevent unwanted reflection signals, and two standard gain horn antennas are placed 1 m away from the sample to satisfy the far field condition. To measure only the reflected signals from the sample, a time gating function in the vector network analyzer was used. Before measuring the sample’s reflection coefficient, we measured a copper plate’s reflection coefficient for measurement reference Γ = −1, with the same dimensions as the sample. To measure the absorption rate for different polarization angles, we rotated the sample with angle ϕ. To measure the absorption rate for different incidence angles, the receiving antenna rotated with angle θ, and the transmitting antenna was placed to satisfy specular reflection. For a metasurface without grating lobes only specular reflection needs to be taken into account.

During the absorption measurements, we covered the inlet and outlet of the microfluidic channel with the plastic tape to prevent evaporation of ethanol. The experiment time for one cycle (e.g., each concentration of ethanol) takes within 3 min, therefore, diffusion effects are not considered in the measurement.

The absorption rate *A*(*ω*) can be calculated from reflection *R*(*ω*) and transmission *T*(*ω*), and is given by:

(3) A(ω)=1−R(ω)−T(ω)

However, the proposed absorber has a metallic sheet on the bottom side, so the EM wave cannot pass through the proposed sensor. Therefore, we can neglect the transmission and calculate the absorption rate by measuring only the reflection coefficient.

**Figure 8:**
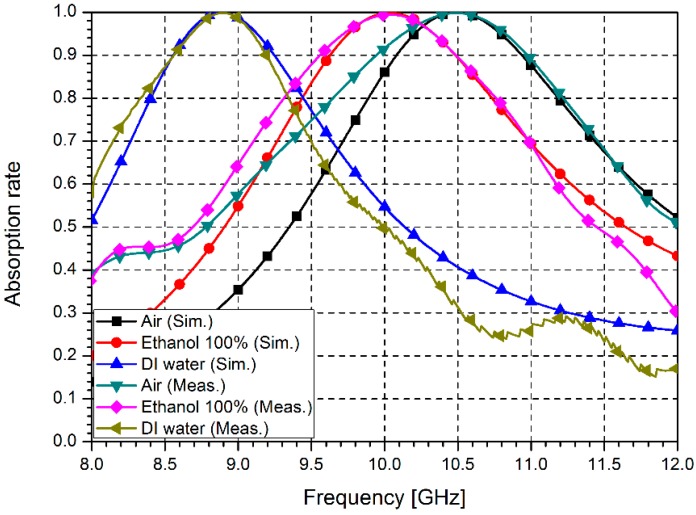
Simulated and measured absorption rates of the proposed metasurface absorber.

Figure 8 shows the simulated and measured absorption rates of the proposed absorber. The proposed absorber exhibits a 99.8% absorption rate at 10.49 GHz in the empty state of the microfluidic channel. When the channel is filled with ethanol, the proposed sensor exhibits a 99.4% absorption rate at 10.04 GHz, and for DI water, the absorption rate is 99.8% at 8.9 GHz. The relatively broader bandwidth is due to low Q-factor of the proposed absorber which results from lossy paper and inkjet printing. Its Q-factor can be improved by PCB and chemical etching process. Nevertheless, inkjet printing technology has benefits of rapid, low-cost, low-temperature, and zero-chemical treatment process.

**Figure 9:**
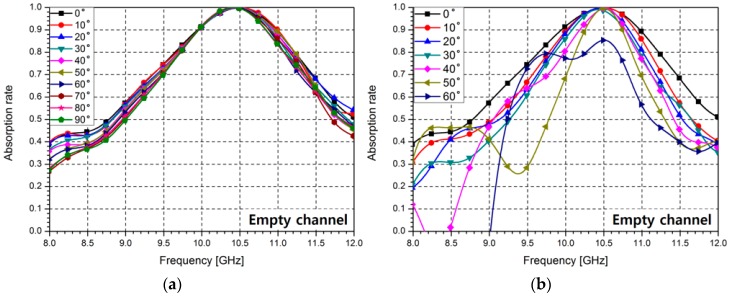
Measured absorption rates of the proposed absorber at different (**a**) polarization angles (ϕ) and (**b**) incidence angles (θ).

The polarization and incident angle dependence of the proposed absorber is also investigated through measurement results. Figure 9a shows the measured absorption rates for different polarization angles (ϕ) of the proposed absorber with empty channels. When the polarization angle is changed from 0° to 90°, the proposed absorber shows almost the same results. Figure 9b shows the measurement results for different incident angles (θ) of the proposed sensor. When the incident angle θ is lower than 50°, the proposed absorber exhibits an over 99% absorption rate at 10.49 GHz, and when the incident angle is 60°, its absorption rate is over 85% at 10.49 GHz.

**Figure 10:**
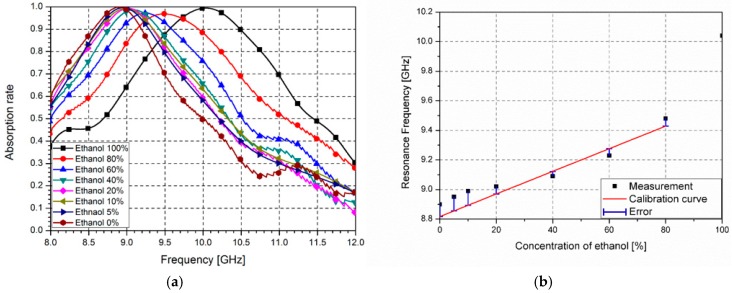
(**a**) Measured absorption rates with different concentrations of ethanol and (**b**) relationship of the resonance frequency and the concentration of ethanol with the calibration curve of *y* = 7.65 × 10^−3^*x* + 8.817 in GHz.

Figure 10a shows the measured absorption rates with different concentrations of ethanol and Figure 10b shows relationship of the resonance frequency and the concentration of ethanol. Ethanol is diluted with DI water. When the ethanol concentration changes from 5% to 100%, the resonance frequency shifts from 10.04 GHz to 8.95 GHz.

In order to see the minimum resolution of the proposed absorber as an ethanol sensor, we measured the absorption ratio for lower concentration of ethanol. Below the ethanol concentrations of 5%, it is very difficult to detect frequency changes. Therefore, 5% concentration is the minimum resolution as the ethanol sensor.

In order to see the linearity of the proposed absorber as the ethanol sensor, the relationship curve of the concentration and resonance frequency is plotted with calibration curves in Figure 10b. The measured relationship is similar to *y* = 7.65 × 10^−3^*x* + 8.817 from 20% to 80% concentrations. Moreover, sensitivity of the ethanol sensor is defined by slope angle of the calibration curve. Therefore, sensitivity of the different concentrations of ethanol is 7.65 × 10^−3^ GHz/percentage. In addition, the proposed absorber using micro fluidic channel has potential as a wireless ethanol sensor application. For instance, the proposed absorber receives a wideband interrogation signal, the resonant frequency can be identified from the reflected frequency response [33]. Therefore, the concentration of ethanol can be monitored wirelessly from a variation of the resonant frequency.

## 4. Conclusions

In conclusion, we have proposed a novel flexible metasurface absorber for large-scale ethanol sensor applications. The proposed metasurface consists of the PDMS channel layer, conductive SRCR pattern layer, and bottom PDMS layer, and each layer is bonded by adhesive laminating film. In order to demonstrate its performance, 15 × 15 unit cells were fabricated. Inkjet-printing technology was adopted to realize the conductive SRCR patterns with silver nanoparticle ink on flexible photo paper. The microfluidic channels were laser etched on the flexible PDMS substrate. The proposed metasurface absorber can detect the concentration of ethanol by measuring the resonance frequency. When the channel is filled with liquid, the effective dielectric constant changes drastically. Therefore, the resonance frequency of the proposed absorber shifts. The experimental results show the resonance frequency increases from 8.9 GHz to 10.04 GHz when the concentration of ethanol is changed from 0% to 100%. The minimum detectable concentration is 5% for ethanol. Sensitivity of the ethanol sensor is 7.65 × 10^−3^ GHz/percentage. For the ethanol concentration, linearity is observed from 2% to 80%.

The proposed metasurface absorber is based on electromagnetic resonance and does not require chemical reaction, any chemical materials are not required for analytical procedures. Due to advantages of microfluidic channels and paper, the proposed absorber is cheap and recyclable for sensor applications. In addition, the proposed microwave sensor can wirelessly detect and monitor chemical materials with low-cost radio-frequency equipment. However, in order to detect the concentration of ethanol, the user must know the type of solution. As a next step, the complete radio-frequency identification (RFID) platform will be developed with analog/digital circuits.
